# The Identification and Evolutionary Trends of the Solute Carrier Superfamily in Arthropods

**DOI:** 10.1093/gbe/evaa153

**Published:** 2020-07-18

**Authors:** Shane M Denecke, Olympia Driva, Hang Ngoc Bao Luong, Panagiotis Ioannidis, Marc Linka, Ralf Nauen, Sven Geibel, John Vontas

**Affiliations:** e1 Institute of Molecular Biology and Biotechnology, Foundation for Research and Technology-Hellas, Heraklion, Greece; e2 R&D Pest Control, Bayer AG, Crop Science Division, Monheim, Germany; e3 Pesticide Science Lab, Department of Crop Science, Agricultural University of Athens, Greece

**Keywords:** insect, transporter, gene family expansion, Computational Analysis of gene Family Evolution, annotation, arthropod

## Abstract

The solute carrier (SLC) transporter superfamily comprises an ancient and ubiquitous group of proteins capable of translocating a range of nutrients, endogenous molecules, and xenobiotics. Although the group has been the subject of intense investigation in both bacteria and mammals, its systematic identification in arthropods has not yet been undertaken. Here, we present a genome-wide identification of all 66 human SLC families in 174 arthropod species. A pipeline (SLC_id) was constructed to identify and group SLCs using a combination of hidden Markov model and BLAST searches followed by filtering based on polypeptide length and the number of transmembrane domains. Comparative analysis of the number of transporters in each family across diverse arthropod lineages was accomplished using one-way analysis of variance (ANOVA) and the Computational Analysis of gene Family Evolution (CAFE). These results suggested that many SLC families have undergone expansions or contractions in particular evolutionary lineages. Notably, the sugar transporting SLC2 family was significantly larger in insects compared with arachnids. This difference may have been complemented by a rapid expansion of the SLC60 family in arachnids which also acts on dietary sugars. Furthermore, the SLC33 family underwent a recent and drastic expansion in aphids, although the biological relevance of this expansion was not possible to infer. Information on specific SLC transporter families across arthropod species can be accessed through an R shiny web application at http://chrysalida.imbb.forth.gr : 3838/Arthropod_SLC_Database/. The present study greatly facilitates further investigation of the diverse group of SLC transporters in arthropods.

**Significance**

The solute carrier (SLC) superfamily is an important group of genes which mediates many important physiological processes. So far, it has not been studied systematically in arthropods. Here, we annotate and compare the SLC superfamily across many arthropod species and identify significant expansions and contractions.

## Introduction

Transmembrane (TM) transporters are one of the most diverse and important protein types in multicellular organisms. They regulate the passage of molecules across biological membranes and provide the metabolic compartmentalization essential for life. One of the largest groups of transporters is the solute carrier (SLC) superfamily (also called the SLC gene series), which is found ubiquitously across the tree of life. The SLC superfamily is not defined by homology but rather functionality; all SLCs transport solutes such as nutrients, ions, and xenobiotics using existing electrochemical gradients or facilitated diffusion without directly hydrolyzing ATP. Individual SLC families, however, are monophyletic, sharing a degree of homology (a rule of 20% identity is used to group family members) and similar substrate specificity (Hediger et al. 2013). There are currently 66 SLC families annotated by the Human Gene Nomenclature committee, which can be further grouped into larger Pfam clans such as the major facilitator superfamily and the amino acid/polyamine/organic cation superfamily.

The molecular biology of nonmodel species can often be driven by studying homologous genes in model systems. Therefore, the classification of genes from nonmodel organisms into SLC families can serve as a starting point for their functional investigation. Variation in the size of SLC families can also suggest evolutionary trends reflecting physiological requirements of organisms while adapting to their environment. These concepts have induced several attempts to systematically identify SLCs in nonhuman species. Hoglund et al. (2011) used a combination of hidden Markov model (HMM) searches and BLAST to identify putative SLCs in a range of species spanning the tree of life. Transporter DB (http://www.membranetransport.org/transportDB2/) has also implemented an in silico pipeline for the identification of all TM transporters (including non-SLCs), which has been applied to thousands of species (Elbourne et al. 2017). Furthermore, the Transporter Classification Database (TCDB) maintains a database of transporter genes classified under a unified nomenclature (Saier et al. 2016). These efforts run in parallel to the continual updating of the SLC transporter family in humans using more sensitive identification algorithms (Perland et al. 2017).

However, these resources all have a notable focus on microorganisms or mammals, and have thus largely ignored other taxonomic groups such as arthropods. Nevertheless, the diversity and adaptive potential of this phylum has attracted the focus of initiatives that aim to sequence large numbers of arthropod genomes and transcriptomes (i5K Consortium 2013; Misof et al. 2014). Many arthropods are also of considerable economic importance. Agricultural pests such as the pea aphid (*Acyrthosiphon pisum*) and the red flour beetle (*Tribolium castaneum*) cause substantial crop damage every year, whereas malaria vectors like *Anopheles gambiae* are considered some of the most deadly species on the planet. On the other hand, insects such as the honeybee (*Apis mellifera*) and the silkworm (*Bombyx mori*) are economically beneficial in the agriculture and textile industries, respectively.

Control of arthropod pests via insecticides and acaricides has been instrumental in limiting their damage, but pesticides frequently have detrimental effects on off-target species such as humans and beneficial insects. Rational pesticide design holds the promise of increased specificity, but this goal remains elusive due to the lack of understanding of insect molecular genetics. This is readily apparent with the SLC superfamily. SLCs have only been systematically identified in *Drosophila melanogaster*, *Aedes aegypti*, and *An. gambiae* (Hoglund et al. 2011; Elbourne et al. 2017). Other studies have annotated individual SLC families, but on an ad hoc basis and often using different nomenclature (Price et al. 2010; Xia et al. 2017; Yang et al. 2017). There have so far been no studies that specifically focus on the SLC superfamily as a whole in any arthropod, and no comparisons have been performed across species.

Better knowledge of the SLC superfamily is necessary for understanding arthropod molecular physiology from both theoretical and applied perspectives. A prerequisite for this is the identification of SLC superfamily members in arthropod species. Here, we present a comprehensive identification of all 66 human SLC families in 174 arthropod species for which good quality protein sets exist. This was accomplished by designing and implementing an in silico pipeline for SLC annotation (SLC_id) which used HMM and BLAST searches followed by filtering based on length and TM domains. Variation among SLC family sizes was then used to determine evolutionary trends over time and ecological niche. This comprehensive data set will be a useful starting point for elucidating functional roles of the SLC superfamily in arthropods.

## Results

### Validation of the Insect SLC Identification Pipeline

**Fig. 1.**
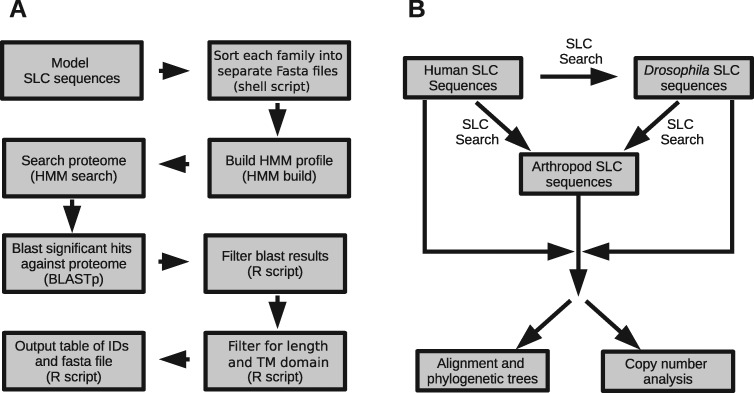
The SLC_id pipeline schematic. (*A*) The SLC_id algorithm starts with “Model Sequences,” representing all SLC transporters in a model species. Through a series of searches and filters, this identifies SLC transporters in a target species and sorts them into families. Parentheses mark the commands or types of scripts used for each step. (*B*) The SLC_id pipeline was then used to identify SLC sequences in arthropods by searching with manually curated sets from *Drosophila melanogaster* and humans.

**Fig. 2.**
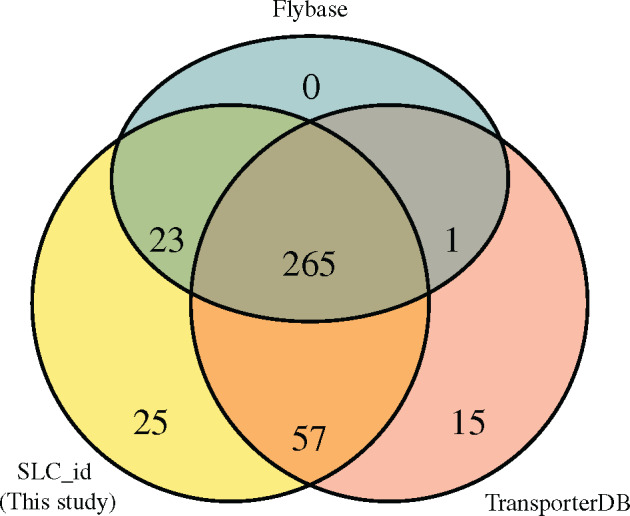
SLC_id validation against *Drosophila melanogaster*. The total number of SLCs identified in *D. melanogaster* by this study (SLC_id; yellow), Transporter DB (pink), and FlyBase (blue) is shown in a Venn diagram. The comparison showed an increase in the amount of transporters detected in our pipeline compared with both transporter DB and FlyBase, but the vast majority of genes were detected in at least two identification strategies.

In order to identify and classify SLC transporters from arthropod gene sets, a pipeline (SLC_id) was constructed which used known SLC transporters in model species as a query to search nonmodel arthropods (fig. 1; see Materials and Methods). The accuracy of the SLC_id pipeline was estimated using manually curated SLC transporters from *Homo sapiens* to search the *D. melanogaster* proteome. A total of 371 SLC transporters were identified in *D. melanogaster*, representing 54/66 of SLC families in *H. sapiens*. This result was compared against two other precompiled sets of *D. melanogaster* SLC transporters: 1) the FlyBase SLC gene group and 2) the secondary transporters from Transporter DB (fig. 2).

All of the 289 SLCs from FlyBase were contained within the SLC_id predicted gene set except for one gene from the SLC7 (cationic amino acid transporter) family *tadr*, which appears to be extremely divergent from any known human SLC7 transporter. Transporter DB predicted 15 genes which were not found in either this study or in FlyBase (supplementary table S1, Supplementary Material online and fig. 2). Four of these 15 genes had human orthologs which have been functionally shown not to be SLC transporters, suggesting that they are falsely annotated. Of the other 11 genes, five were orthologous to human genes which have been suggested to be SLCs (Perland et al. 2017), but have not been categorized into SLC families. Thus, these were not included in the initial human SLC transporters used in the SLC_id pipeline. The remaining six genes predicted uniquely by transporter DB contained either Major facilitator superfamily or SLC6 domains, suggesting that they may be uncharacterized SLC transporters. About 25 *D. melanogaster* genes were predicted uniquely by the SLC_id. Of these five had been functionally characterized as solute transporters in *D. melanogaster*, in line with this study’s annotation (references in supplementary table S1, Supplementary Material online). The remaining 20 genes all had human orthologs predicted on FlyBase which fell into their corresponding SLC family predicted by SLC_id. These data suggest that the SLC_id pipeline was sensitive enough to identify SLCs within a distantly related species, while avoiding any clear false positives.

### SLC Family Size Variation across Different Taxonomic Groups and Diet Types

After validating the SLC_id pipeline, we then sought to annotate the SLC transporter families in 192 other nonmodel arthropod species sampled from different lineages (supplementary fig. S1, Supplementary Material online). 19 of these had BUSCO completeness scores of <75 and were discarded to avoid distortions due to poor proteome quality. Of the remaining 173 arthropods, the total number of SLC transporters ranged from a low of 193 in the itch mite *Sarcoptes scabiei* to a high of 610 in the horseshoe crab *Limulus polyphemus*; this is likely the result of a recent genome duplication in the Xiphosura lineage (supplementary fig. S2, Supplementary Material online; Kenny et al. 2016).

**Fig. 3.**
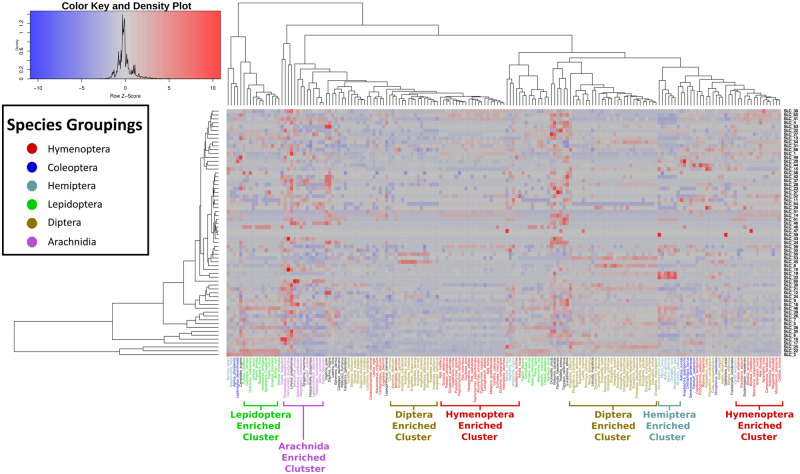
Clustering of species by SLC count. Species (*x*-axis) were hierarchically clustered based on their SLC family sizes (*y*-axis) in order to see whether evolutionarily related species showed similar SLC profiles. Species are color coded according to their taxonomic group (blue, Tribolium; red, Hymenoptera; brown, Diptera; magenta, Hemiptera; purple, Arachnida; green, Lepidoptera; black, other). Boxes toward the red end of the spectrum have a relatively large family, whereas bluer boxes have lower copy numbers. Many related species cluster closely together, but other areas of the graph are relatively diverse in terms of their composition.

We further calculated the size of each SLC family in each arthropod species with the aim of determining how diet type and evolutionary lineage influenced family size (supplementary table S2, Supplementary Material online). Hierarchical clustering of species according to SLC family sizes showed that species predominantly grouped by evolutionary lineage, but that this grouping was imperfect (fig. 3). For example, almost all of the Lepidoptera clustered closely together, whereas other groups such as Hymenoptera and Diptera split into subclusters*.* Apart from a slight clustering of carnivores, there was no coherent grouping of species by either of the dietary categories of “phagy” (e.g., monophagous, polyphagous) or “vory” (e.g., herbivore, omnivore) (supplementary fig. S3, Supplementary Material online).

**Fig. 4.**
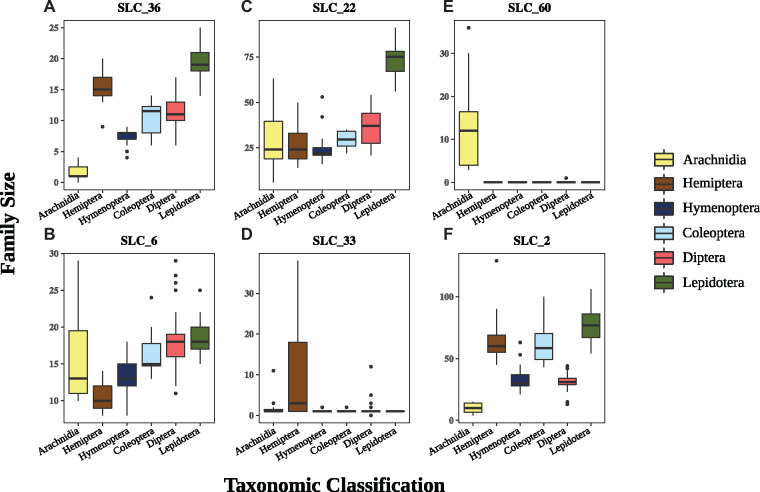
Variation in SLC family size among taxonomic groups. The *x*-axis of each boxplot shows the six different taxonomic groups represented by at least eight species in our data set. The *y*-axis shows the family size in a given family (A, SLC36; B, SLC22; C, SLC2; D, SLC6; E, SLC60; F, SLC33). The box of each plot is represented by quartiles 1 and 3, whereas the lines represent 1.5 times the quartile range. Outliers are represented by black dots.

These findings were further explored by performing an analysis of variance (ANOVA) where the impact of taxonomic group and diet were evaluated for each SLC family size. 14 significant relationships were found (table 1), several of which are particularly noteworthy. The amino acid transporting SLC36 (proton-coupled amino acid) and SLC6 (sodium and chloride dependent neurotransmitter) families showed variable levels across taxonomic groups. The former was significantly larger in Lepidoptera and Hemiptera and the latter was smaller in Hemiptera (fig. 4*A* and *B*). The SLC22 (Organic cation/anion/zwitterion transporter) family was also larger in Lepidoptera, but relatively stable in size across other taxa (fig. 4*C*). The SLC33 (acetyl-CoA transporter) family also showed a striking expansion in Hemiptera (fig. 4*D*). Lastly, two families of sugar transporters showed highly variable patterns. The SLC2 (Facilitative GLUT) family of sugar transporters, showed low numbers in Diptera, Hymenoptera, and Arachnida, but higher numbers in Coleoptera, Hemiptera, and Lepidoptera (fig. 4*E*). The very low number of SLC2 sugar transporters in Arachnida is interesting given that a striking expansion in the SLC60 (Glucose transporter) family has taken place in arachnids, while being almost completely absent in Insecta (fig. 4*F*).

**Table 1:** This Table Shows the Most Significant Outcomes of the One-Way ANOVA Comparing SLC Family Size against “Vory,” “Taxonomic Group,” and “Phagy”

Family	Covariable	*P* Value	Bonf	Maximum Effect
SLC_36	Taxonomic_Classification	7.98E-56	1.44E-53	17.46
SLC_2	Taxonomic_Classification	1.04E-45	1.88E-43	67.33
SLC_46	Taxonomic_Classification	8.60E-43	1.55E-40	9.77
SLC_22	Taxonomic_Classification	5.02E-41	9.04E-39	42.98
SLC_18	Taxonomic_Classification	1.05E-29	1.90E-27	8.45
SLC_60	Taxonomic_Classification	8.13E-28	1.46E-25	13.45
SLC_13	Taxonomic_Classification	2.99E-21	5.38E-19	4.30
SLC_63	Taxonomic_Classification	3.60E-20	6.47E-18	4.32
SLC_35	Taxonomic_Classification	4.39E-19	7.90E-17	9.04
SLC_6	Taxonomic_Classification	9.96E-19	1.79E-16	8.45
SLC_19	Taxonomic_Classification	1.62E-17	2.91E-15	5.29
SLC_12	Taxonomic_Classification	4.27E-15	7.68E-13	4.58
SLC_16	Taxonomic_Classification	3.76E-13	6.76E-11	19.47
SLC_33	Taxonomic_Classification	8.86E-13	1.59E-10	8.74

Note.—The only significant comparisons were found in the “taxonomic group” comparison type.

Lastly, we calculated the coefficients of variation for each family with the aim of describing families that were particularly stable across the species examined in our study. The SLC25 (Mitochondrial carrier) was the least variable despite being one of the largest families (supplementary table S3, Supplementary Material online). The next most stable family was the SLC7 family, despite being significantly higher in Hemiptera (supplementary fig. S4, Supplementary Material online). Other stable families include SLC30, SLC12, and SLC24 families which are all involved in ion transport and homeostasis (supplementary table S3, Supplementary Material online).

### Detailed Analysis of Family Expansion with CAFE

**Fig. 5.**
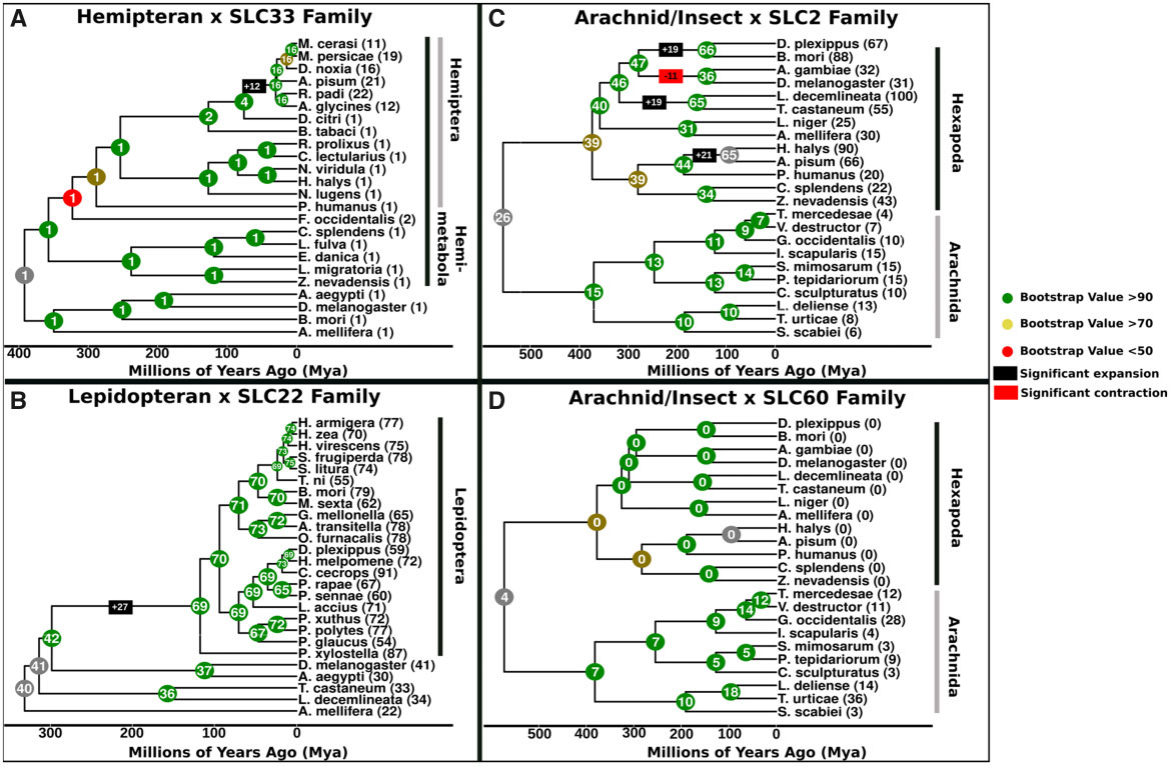
Phylogenetic analysis of SLC family size variation. CAFE was used to analyze the evolutionary fates of selected SLC families. (*A*) A hemipteran enriched tree considering the SLC33. (*B*) An arthropod-wide tree with SLC2 and an Arachnida enriched tree with (*C*) SLC35 and (*D*) SLC60. The color of each node corresponds to the bootstrap support of each node (red, <80; yellow, >80 and <90; green, >90; gray, NA). Numbers in parentheses next to each species represent the detected number of SLC family members in each species. All trees were calibrated for time and are plotted in terms of millions of years ago (Ma). Black and red boxes represent expansions supported by at least two species.

In order to provide a more detailed picture of SLC family expansion and contraction, Computational Analysis of gene Family Evolution (CAFE) was employed. This analysis gave greater resolution to the differences observed between taxonomic orders by estimating the size of each SLC family along a phylogenetic tree (supplementary table S4, Supplementary Material online). We focused on the SLC33 acetyl-CoA transporting family, the organic ion transporting SLC22 family, and on two families of dietary sugar transporters (SLC2, SLC60). The expansion of SLC33s in Hemiptera (fig. 4*A*) was found to have taken place specifically in aphids, and all other Hemiptera had only one or two family members (fig. 5*A*). In contrast, the expansion of SLC22 transporters in the Lepidoptera clade (fig. 4*C*) was predicted to have taken place when Lepidoptera diverged from Diptera (fig. 5*B*). Particularly interesting is the case of SLC2 and SLC60 transporters. The SLC60 family is absent in almost all hexapods, whereas it is found in varying numbers among arachnids (fig. 5*D*). The SLC2 family shows a largely opposite trend. Arachnids appear to have contracted their SLC2, whereas there is a general expansion in hexapods. Some lineages such as Hemiptera, Coleoptera, and Lepidoptera have undergone additional expansions in this family (fig. 5*C*).

### Data Access and Availability

**Fig. 6.**
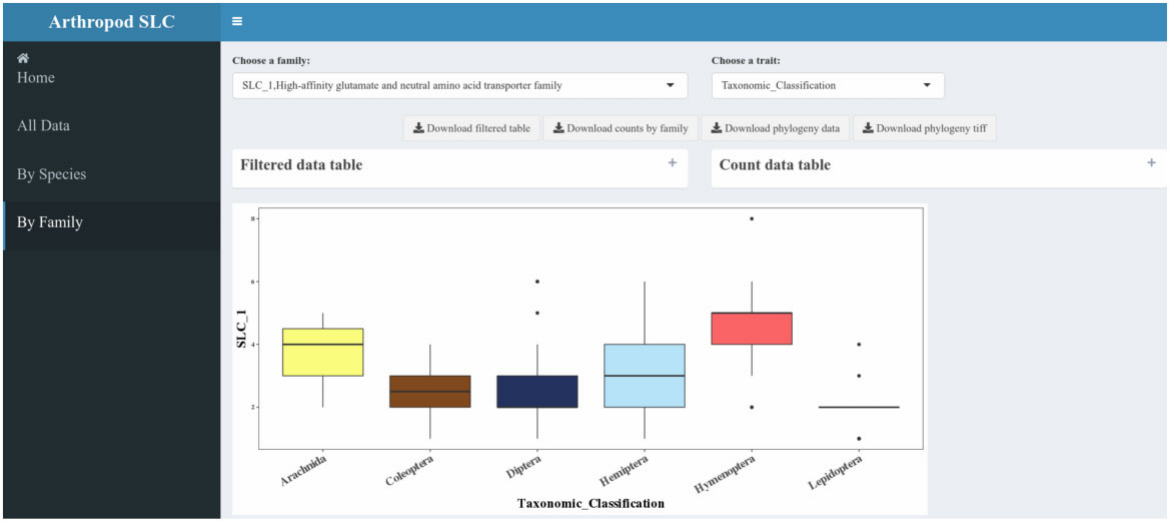
SLC_id Shiny application. The Arthropod SLC Database serves as an access portal to the data presented in this study. Users can download plots, tables, and Fasta sequences by species or family. The database can be found at http://chrysalida.imbb.forth.gr : 3838/Arthropod_SLC_Database/.

In order to make the findings of this work more accessible and to provide a public resource for other research groups working on arthropod SLC transporters, we set up a web application containing data associated with this paper in R Shiny (http://chrysalida.imbb.forth.gr : 3838/Arthropod_SLC_Database/; supplementary file S1, Supplementary Material online). The database features search bars and dropdown menus to retrieve information on either a particular species or a given SLC family (fig. 6). In the “Species” tab, selecting a given species will display a table showing all SLC transporters in that species, with a public identifier (e.g., Uniprot or NCBI) and the family to which each belongs. Another table shows the size of each SLC family in the selected species, and a histogram indicates how the total number of SLC transporters in that species compares with other arthropods. In the “Family” tab, selection of a given SLC family will provide tables containing information about all SLC transporters from that family broken down by species. Additionally, an interactive plot shows how the different comparison types (e.g., taxonomic group and phagy) vary within that family. Lastly, a phylogeny of the chosen SLC family will be displayed including the transporters in *H. sapiens* and five of the best-studied arthropod species: *D. melanogaster*, *T. castaneum*, *B. mori*, *Ac. pisum*, and *Ap. mellifera.* All SLC amino acid sequences can be freely downloaded in Fasta format along with images of all figures and the Newick formatted data used to generate all phylogenetic trees. It is hoped that the easy access to SLC data provided by this app will stimulate the functional characterization of these genes by nonspecialists.

## Discussion

### SLC_id Pipeline Validations

In order to verify that the SLC_id pipeline did not predict excessive amounts of false positives or false negatives, the SLCs predicted in this study were compared against previously published data sets from *Drosophila* (fig. 2). The majority of the 15 transporters predicted uniquely by TransporterDB seem to be bona fide transporters, but show homology to human genes not included in the current SLC classification system (supplementary table S1, Supplementary Material online). These were not included in the original *H. sapiens* SLC set used in the SLC_id pipeline and therefore were not expected to be identified in our analysis. Also of note were the 25 *Drosophila* SLCs identified only in this study. Many of these may be attributed to differences in the range of SLC families sets considered in each search. For example, SLC_id includes all 66 families named in the Human Gene Nomenclature Committee and SLC Tables, whereas FlyBase only has SLC families 1–50. Unfortunately, annotations from other arthropods are largely absent from the literature, making it impossible to benchmark the SLC_id strategy more extensively. Only *Ae. aegypti* and *An. gambiae* were annotated using both Transporter DB and SLC_id which showed −5% and +2% differences in total SLC numbers between the two algorithms. However, a more detailed comparison could not be performed due to different protein databases used in each pipeline.

These data suggest that the pipeline presented in this study is a valid way to predict SLC transporters and even classify them into one of the known SLC families. Although only tested in arthropods, the SLC_id pipeline could be used to identify SLC transporters in more distantly related species as only the use *Drosophila* of as a model species is arthropod specific. However, this awaits testing and validation against other transporter databases.

### SLC Family Size Variation among Taxonomic Groups

Among the 63,329 transporters identified across 174 arthropods, (*Dr*o*sophila* plus 192 nonmodel arthropods minus the 19 species excluded for quality) several of the SLC families showed dramatic expansions or contractions. Such changes in the size of other gene families have previously been associated with functional adaptation (e.g., Sharpton et al. 2009), which led us to test how dietary traits like vory (herbivore and carnivore) or phagy (oligophagous, polyphagous) correlated with SLC family sizes. However, no significant relationship between any SLC families and these dietary traits was found, unlike families detoxification enzymes such as cytochrome P450s, carboxylesterases, and glutathione-S-transferases (Rane et al. 2016, 2019). Grouping by evolutionary lineage (taxonomy) was a far better predictor of SLC family size. Although there were many interesting individual species included in our analysis (e.g., the myriapod *Strigamia maritima*), one must be cautious making inferences from small numbers of species. The quality of the genome annotation can bias the predicted SLC family size in either direction, so we have restricted our focus to evolutionary trends which are supported by many species.

Of the five major families implicated in dietary sugar transport (SLC2, SLC5, SLC45, SLC50, and SLC60), two (SLC2 and SLC60) showed significant amounts of variation among taxonomic groups. Although the SLC2 was the predominant family of sugar transporters in insects (particularly winged insects), arachnids showed expansions of the SLC60 family (figs. 4*E* and *F* and 5*C* and *D*). The SLC2 sugar transporters have been the best characterized and are known to transport nutrient sugars such as hexoses and polyols in mammals (Mueckler and Thorens 2013). The other families are less well-studied, but the SLC60 family has been shown to act on sugars such as glucose and fructose in mammalian kidneys (Horiba et al. 2003). The differences in the family sizes between insects and arachnids are suggestive of a compensatory mechanism whereby a lineage lacking one sugar transporting family is complemented by an expansion of the other family (fig. 5*C* and *D*). This compensatory hypothesis is particularly compelling with the SLC2 and SLC60 situation as: 1) the expansions in these families are very clear (fig. 4) and 2) both families have been characterized as acting on dietary nutrients such as glucose and fructose. A similar situation is seen with the SLC6, 7, and 36 amino acid transporters. The SLC6 family is underrepresented in Hemiptera, whereas the latter two have undergone expansions in agreement with previous studies (Dahan et al. 2015; Denecke et al. 2020; supplementary fig. S3, Supplementary Material online and fig. 4). However, the presence of some SLC2 family members in arachnids and SLC6 members in Hemiptera highlights that this hypothesis must be treated as suggestive until functional work on these families confirms or rejects thiese hypotheses.

Less clear is the role of the SLC33 and SLC22 families, which are significantly larger in Hemiptera and Lepidoptera, respectively. The SLC33 family is thought to transport acetyl-CoA into the endoplasmic reticulum, allowing for the acetylation of glycoproteins and gangliosides (Hirabayashi et al. 2013). Additionally, the SLC33 protein was linked to drug transport and resistance in *Plasmodium falciparum* (Lim et al. 2016). The lack of information on this family makes it difficult to form a precise hypothesis on what (if any) adaptive role the SLC33 family expansion may play in aphids. The SLC22 family has been more thoroughly studied and has been linked to drug transport in humans (Estudante et al. 2016). Although the fitness benefits (if any) underpinning this expansion can only be hypothesized here, it is noted that Lepidoptera larvae encounter a wide range of plant secondary metabolites in their lifespan owing to their choice of host and quantity of food consumed. However, it must also be noted that the SLC22 family is extremely complex, and many members likely play endogenous roles apart from xenobiotic transport.

### Future Uses of the SLC Data

Due to space limitations, this work has necessarily had to focus on a subset of evolutionary trends in specific SLC families. However, it is hoped that this data set will be a starting point for a fuller characterization of SLC transporters in insects. It was for this reason that a web application (http://chrysalida.imbb.forth.gr : 3838/Arthropod_SLC_Database/) was created to facilitate access to, and exploration of, the data. Furthermore, the *SLC_id.sh* script can be downloaded (https://github.com/shanedenecke/SLC_ID_SCRIPTS.git) and used to search the genomes of newly annotated arthropods.

Although other more comprehensive transporter databases exist (e.g., TransporterDB and the TCDB), our focus on arthropod SLC transporters fills a gap that has so far not been adequately addressed. The SLC_id pipeline and data set should therefore be seen as complementary to these existing resources rather than being a competitor or replacement.

The characterization of SLC transporters must be supported by functional studies performed for each transporter individually. Although homology (family membership) can suggest function, members of the same families often have quite different molecular properties. For example, members of the SLC12 (electroneutral cation-coupled Cl) transporter family act as ion exchangers, but the stoichiometry and substrate specificity vary between members of the family and between closely related species (Kalsi et al. 2019). Luckily, there exists a variety of methodologies to functionally characterize SLC transporters. Because most SLCs are energized by electrochemical gradients, the *Xenopus* oocyte system has become a widespread tool, but others, such as cell-based assays or in vivo assays, are also feasible (Weinglass et al. 2008; Wang et al. 2018).

One area particularly deserving attention is the role of SLC transporters in the transport of pesticides across epithelia, such as the midgut (Denecke et al. 2018). In mammals, a role for SLCs in drug transport is well established and widespread (Estudante et al. 2016; Girardi et al. 2020), and connecting insect transporters with this literature informed the decision to use SLC nomenclature in this study (as opposed to the TCDB codes). The involvement of insect SLCs xenobiotic transport is also supported by several studies which have associated individual SLC genes or families with resistance to either pesticides or plant secondary metabolites (Torrie et al. 2004; Dermauw et al. 2013; Schmidt et al. 2019). Such associations can be expanded by reverse genetic tools such as CRISPR-Cas9 and RNAi that are now widely applicable in insects. It is thus hoped that future work on SLC transporters in insects can build on the data presented here and the vast amount of human literature to address concepts like pesticide transport from a genetic perspective.

## Materials and Methods

### Databases and Sequences Used

Amino acid sequences for each member of the manually curated human SLC superfamily were downloaded from Uniprot using gene codes derived from the combined resources of *SLC Tables* (http://slc.bioparadigms.org/) and the Human Gene Nomenclature Committee (https://www.genenames.org/data/genegroup/#!/group/752), comprising a total of 66 SLC families and 434 genes. Proteomes from 167 arthropod species were downloaded from OrthoDB v10 (Kriventseva et al. 2019), or alternative publicly available resources (supplementary table S2, Supplementary Material online). These contained only one amino acid sequence per gene, representing the longest isoform. Only *Nezara viridula* was gathered from a de novo-assembled transcriptome (Denecke et al. 2020; NCBI Bioproject PRJNA557118). In order to compare this pipeline to other SLC data sets, *D. melanogaster* SLCs identified in this study were compared with two existing *D. melanogaster* SLC data sets derived from Transporter DB (Elbourne et al. 2017) and FlyBase (Attrill et al. 2016). The SLC3 family was excluded from our analysis as it is known to share close homology with a group of insect α‐glucosidase enzymes (Gabriško and Janeček 2009).

### The SLC_id Search Pipeline

A comprehensive SLC identification pipeline (SLC_id) was designed, which used the SLC transporter set of a model species (*H. sapiens* or *D. melanogaster*) to identify and classify SLC transporters in nonmodel proteomes and then classify them into families (fig. 1*A*). The pipeline relied on a concept of HMM searches followed by reciprocal BLAST that has been previously used to identify SLC transporters in distant lineages (Hoglund et al. 2011). First, amino acid sequences from each SLC family in the model species were used to create HMM profiles using the HMMER package v3.2.1 (Eddy 2011). These profiles were then used to search the target proteomes for candidate SLCs using the default significance threshold (*P* < 0.01). These candidates were then filtered based on a number of specific criteria (described below) to determine whether the gene was a true SLC transporter and to classify it in a particular family.

Candidate SLC transporters were used as queries in a protein BLAST (BlastP) against the proteome of the original model species using default parameters (evalue threshold at 10), which gave maximum sensitivity. Candidates were only considered part of a specific SLC family if they met the following criteria. 1) The top BLAST hit must be a member of the family. 2) At least four out of the top five BLAST hits must be a part of the family, or all family members must be present if the family has less than five total members. 3) The percent identity of the top hit must be >20% in accordance with generally accepted SLC family membership (Hediger et al. 2013). SLC candidates not meeting these criteria were not sorted into families. Candidates showing four out of the top five hits corresponding to different SLC families were called as “Unsorted.” If the top BLAST hit was overwhelmingly significant compared with the next most significant hit (>10^30^-fold change in E value), the candidate was considered a part of that family even if it did not meet the other conditions.

Candidates that passed the BlastP filter were then assessed based on their amino acid length and number of TM domains. For each SLC family, all curated sequences from both human (http://slc.bioparadigms.org/) and *D. melanogaster* (Attrill et al. 2016) were used to construct minimum and maximum lengths for each SLC family. Candidate SLCs were removed from the analysis if they were 1/3 smaller than the minimum length or 1/3 longer than the maximum length. TMHMM (Krogh et al. 2001) was used to calculate the number of TM domains present in each SLC gene from the two model species, and a minimum threshold for each family was set at half the lowest number of domains detected in that family. Candidate SLC transporters with lengths outside their family’s length range or with too few TM domains were discarded.

### Identification of Arthropod SLC Transporters

In order to identify SLC transporters in nonmodel arthropods, SLC transporters from *H. sapiens* and *D. melanogaster* were used to search nonmodel arthropod UniGene (one sequence per gene) proteomes (fig. 1*B*). The SLC_id pipeline was first tested on the *D. melanogaster* proteome using the high-quality, manually curated *H. sapiens* SLC genes as a reference. The SLCs from both *H. sapiens* and *D. melanogaster* were then used as queries to search the genomes of 170 other arthropod species. Candidates identified in either the *D. melanogaster* or *H. sapiens* search were considered as candidate SLCs for that species. As the proteomes of some nonmodel arthropods may contain many fragmented genes, we estimated the completeness of each proteome using BUSCO (Waterhouse et al. 2019). Species with less than a 75% completeness score were removed from the analysis.

### SLC Family Phylogenetic Trees

In order to better visualize relationships between identified transporters and predict their putative function, phylogenetic trees were constructed for every SLC family separately. This analysis included some of the most well-studied arthropod species such as *D. melanogaster, Ap. mellifera, B. mori, T. castaneum*, and *Ac. pisum*, with *H. sapiens* as a nonarthropod outgroup. For each SLC family, amino acid sequences from all species were aligned with MAFFT (Katoh and Standley 2013) with the L-INS-i settings and trimmed with Trimal (Capella-Gutiérrez et al. 2009) using the “automated1” algorithm. Lastly, a maximum likelihood phylogenetic tree was inferred with RAxML 8.20 using parameters “-f a” for rapid bootstrap analysis, “-p 12345 and -x 12345” for a random number seed and “-N 500” for 500 bootstraps (Stamatakis 2014). All trees were visualized with the ggtree package in R (Yu et al. 2017).

### Family Size Variation Analysis

The number of distinct genes in each family (family size) was compared among different groups of species based on their taxonomy and their diet. Groups were excluded from the analysis if there were not at least eight members present in the data set. Categorization for each comparison type generated a table of species data (supplementary table S2, Supplementary Material online); this was heavily based on a previously published data set (Rane et al. 2019), with some additions and manual edits. Significant differences in SLC family size was established with one-way ANOVA using the Bonferroni correction. Comparisons with a *P* value <10^−5^ and effect sizes of over three genes were considered significant.

### Computational Analysis of Gene Family Evolution

In order to estimate the rate of SLC gene evolution across evolutionary time, version 5 of CAFE was employed (De Bie et al. 2006; Han et al. 2013). CAFE requires family counts representing the number of genes in each family in each species and an ultrametric phylogenetic tree as inputs. Family counts were provided by the SLC_id pipeline as described earlier. Three separate ultrametric trees were generated for a subset of species used here representing Lepidoptera, arachnids/insects, and Hemiptera (supplementary table S5, Supplementary Material online). First, phylogenomic trees were generated by extracting all 1:1 orthologs for each species using OrthoFinder under default settings (Emms and Kelly 2019). These phylogenies were then normalized using the “chronos” function of the “ape” R package using a discrete model, and were time-calibrated using several known evolutionary divergences published previously (Misof et al. 2014; supplementary table S6, Supplementary Material online). CAFEv5 was then run using the automatically calculated λ value, which yielded an estimate of SLC family size for each node of the tree.

### Arthropod SLC Database and Data Availability

In order to facilitate user access for nonspecialists, a database for Fasta sequences and tables with external reference codes (Uniprot or NCBI) was created using the R shiny interface (http://chrysalida.imbb.forth.gr:3838/Arthropod_SLC_Database/; Chang et al. 2020). All scripts used to analyze the data were written in Python, R, or shell (Bash) and are available on GitHub at (https://github.com/shanedenecke/SLC_id_scripts).

## Data Availability

All raw sequencing data can be downloaded from OrthoDB (https://www.orthodb.org/) or NCBI (https://www.ncbi.nlm.nih.gov/). Furthermore, all processed data can be accessed at http://chrysalida.imbb.forth.gr:3838/Arthropod_SLC_Database/.

## Supplementary Material

evaa153_Supplementary_Data

## Notes

**Data deposition:** This project has been deposited at http://chrysalida.imbb.forth.gr:3838/Arthropod_SLC_Database/.
