# Supplementary material for: The Identification and Evolutionary Trends of the Solute Carrier Superfamily in Arthropods
Source: Genome Biol Evol. 2020 Jul 18;12(8):1429–39. doi: 10.1093/gbe/evaa153 (PMC7487162; doi:10.1093/gbe/evaa153)
Supplement: evaa153_Supplementary_Data [file evaa153_supplementary_data.zip › Legends suppl materials.docx]

*Figure S1:*

All species included in the study were grouped according to their taxonomic group and displayed in a histogram. The largest number of species was in *Diptera* while other common insect orders were also well represented.

*Figure S2:*

A histogram is shown displaying the total number of SLC transporters in each species. Totals ranged from 166 to 565. Two apparent peaks were present in the dataset: one between 270-330 and another centered around 360. These peaks can be partially explained by the large number of *Drosophila* and *Anopheles* species present in the dataset.

*Figure S3:*

This figure is almost identical to Figure 4, but with species highlighted according to their diet type rather than taxonomic grouping. Panel A shows highlighting according to their *phagy* (e.g. polyphagous) and panel B is colored according to *vory* (e.g. herbivore).

*Table S1:*

A table is shown displaying the unique genes identified in Transporter DB and in our study. Corresponding information on each gene including the predicted gene family and whether the gene has been functionally verified.

*Table S2:*

All species 193 arthropod species which were analysed in this study are displayed with their corresponding metadata. This includes taxonomic information (e.g. Order), diet information (e.g. phagy), the source of the UniGene set, and the family sizes for all SLC families.

Table S3:

Coefficients of variance were calculated for each SLC family across the species considered in this study.

*Table S4:*

The family sizes of each SLC gene family is displayed for each species. The tree in .nwk format is presented in the first row and the nodeIDs for that tree are presented in the 2^nd^. All remaining rows contain the number of SLC transporters in each family that were measured in our study or predicted by CAFE (in the case of nodes between groups).

*Table S5:*

A table is shown with every arthropod species used in our study and information on their classifications in terms of *vory, taxonomic group,* and *phagy.*

*Table S6:*

A subset of calibration points for phylogenetic trees were taken from Misof *et. al.* 2014 and used to create the x-axis of the CAFE trees.
