# Supplementary figures and images for: The Identification and Evolutionary Trends of the Solute Carrier Superfamily in Arthropods
Source: Genome Biol Evol. 2020 Jul 18;12(8):1429–39. doi: 10.1093/gbe/evaa153 (PMC7487162; doi:10.1093/gbe/evaa153)

**Frequency**

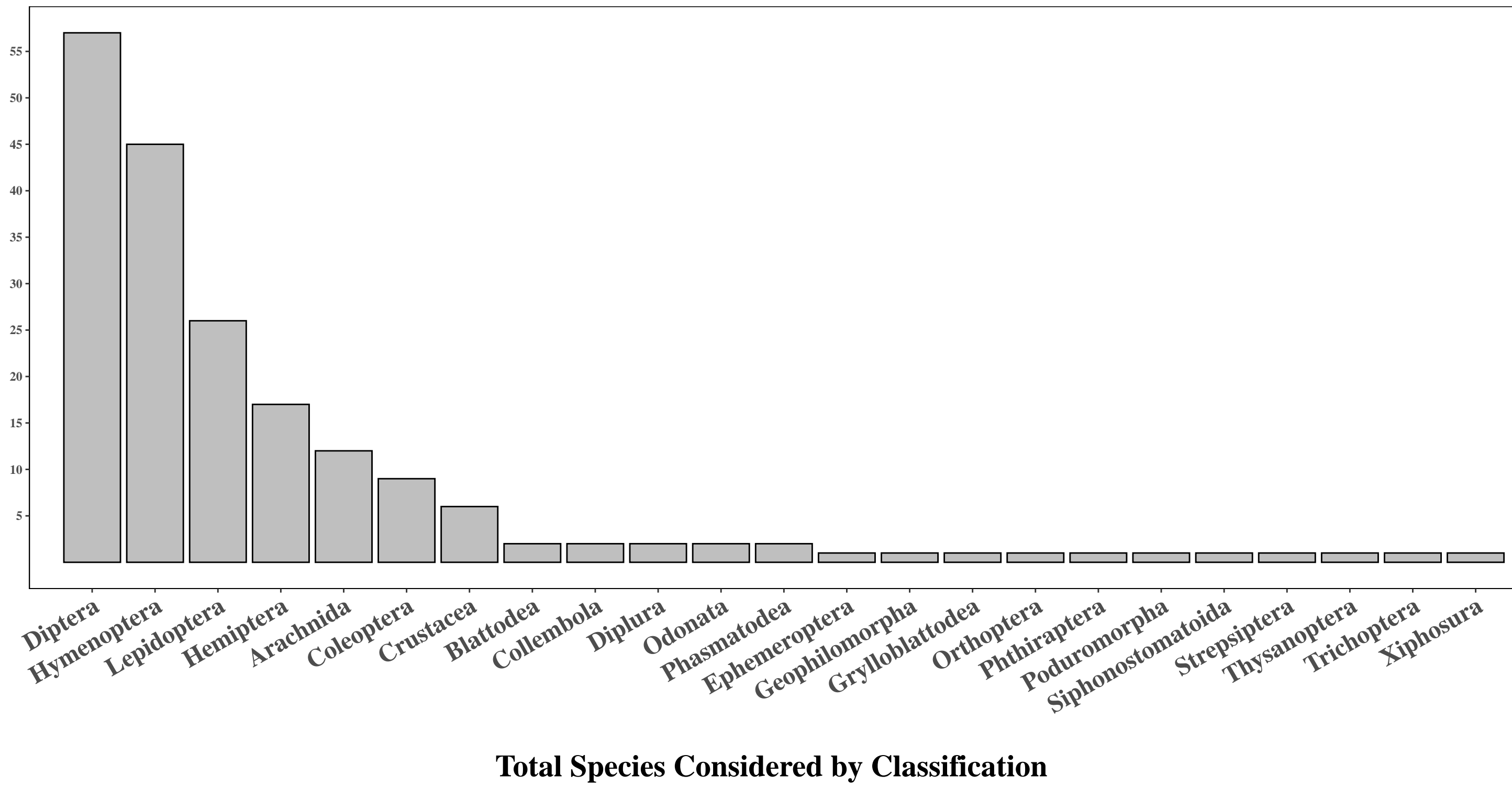

Supplement: evaa153_Supplementary_Data [file evaa153_supplementary_data.zip › FigureS1_Taxonomy_counts_histogram.pdf]

**Frequency**

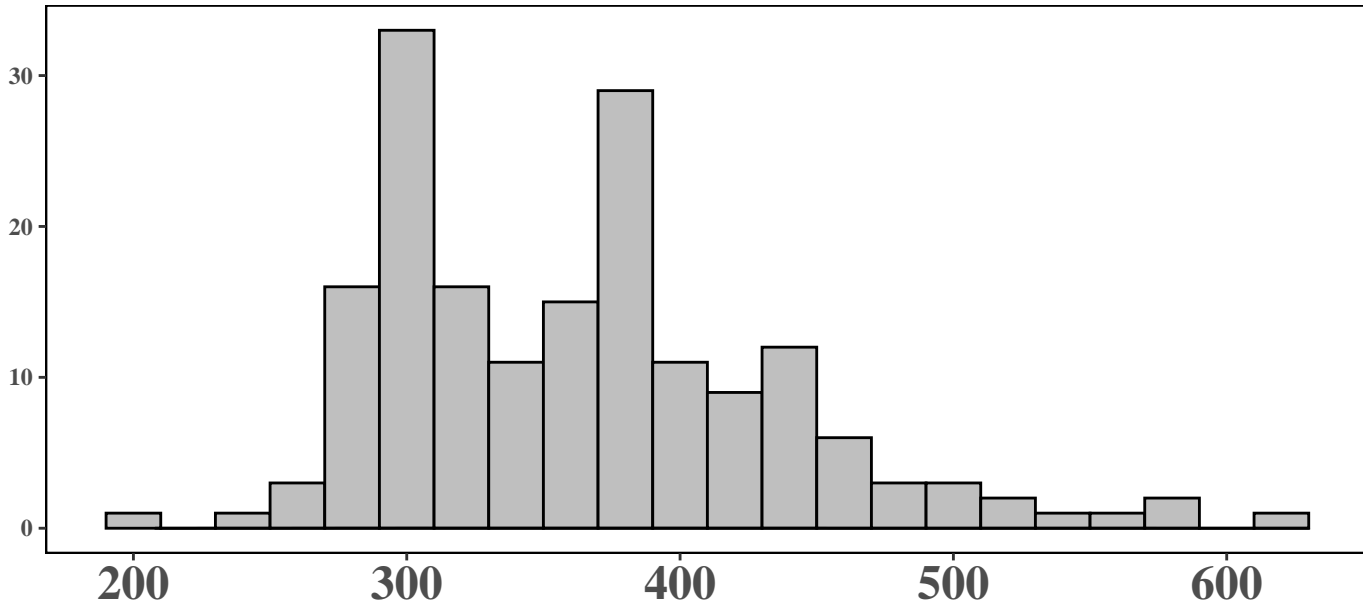

**Total SLCs Identified in Species**

Supplement: evaa153_Supplementary_Data [file evaa153_supplementary_data.zip › FigureS2_SLC_counts_histogram.pdf]

SLC\_7

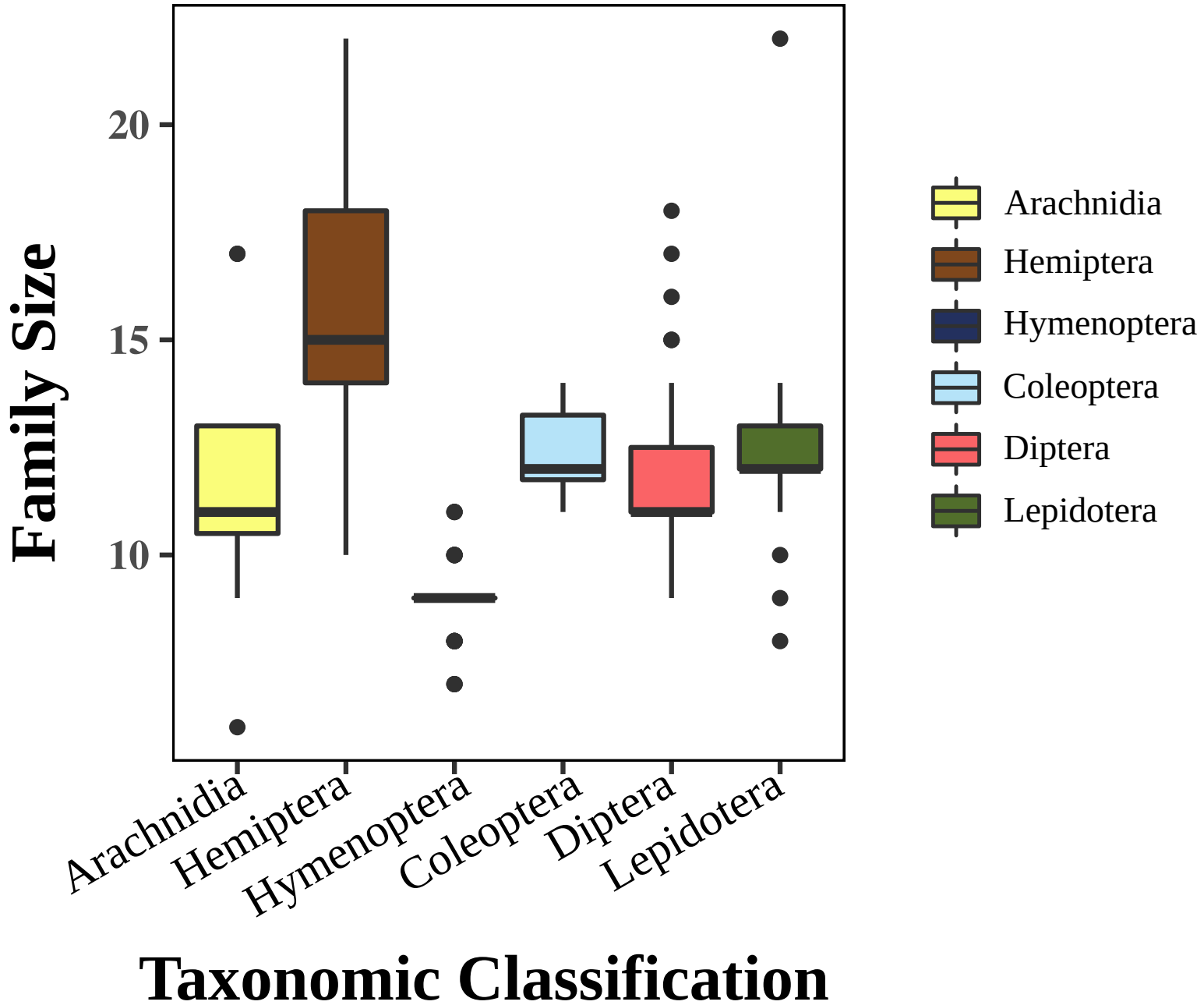

Supplement: evaa153_Supplementary_Data [file evaa153_supplementary_data.zip › FigureS3_Taxonomic_Classification_SLC7.pdf]
